# Insulin sensitivity index (ISI_0, 120_) potentially linked to carbon isotopes of breath CO_2_ for pre-diabetes and type 2 diabetes

**DOI:** 10.1038/srep11959

**Published:** 2015-07-07

**Authors:** Chiranjit Ghosh, Prabuddha Mukhopadhyay, Shibendu Ghosh, Manik Pradhan

**Affiliations:** 1 Department of Chemical, Biological and Macromolecular Sciences, S. N. Bose National Centre for Basic Sciences, Salt Lake, JD Block, Sector III, Kolkata-700098, India; 2 Department of Medicine, Vivekananda Institute of Medical Sciences, 99 Sarat Bose Road, Kolkata-700027, India; 3 Department of Medicine, Raipur Institute of Medical Sciences, Raipur-492006, Chhattisgarh, India

## Abstract

New strategies for an accurate and early detection of insulin resistance are important to delay or prevent the acute onset of type 2 diabetes (T2D). Currently, insulin sensitivity index (ISI_0,120_) is considered to be a viable invasive method of whole-body insulin resistance for use in clinical settings in comparison with other invasive sensitivity indexes like homeostasis model assessment (HOMA), and quantitative insulin sensitivity check index (QUICKI). To investigate how these sensitivity indexes link the ^13^C/^12^C-carbon isotopes of exhaled breath CO_2_ to pre-diabetes (PD) and type 2 diabetes in response to glucose ingestion, we studied excretion dynamics of ^13^C/^12^C-isotopic fractionations of breath CO_2_. Here, we show that ^13^C/^12^C-isotope ratios of breath CO_2_ were well correlated with blood glucose, insulin, glycosylated-hemoglobin as well as with HOMA-IR and 1/QUICKI. Conversely, the strongest correlation was observed between 1/ISI_0,120_ and breath CO_2_ isotopes. Consequently, we determined several optimal diagnostic cut-off points of 1/ISI_0,120_ and ^13^CO_2_/^12^CO_2_-isotope ratios to distinctively track the evolution of PD prior to the onset of T2D. Our findings suggest that isotopic breath CO_2_ is a novel method for accurate estimation of ISI_0,120_ and thus may open new perspectives into the isotope-specific non-invasive evaluation of insulin resistance for large-scale real-time diabetes screening purposes.

Type 2 diabetes mellitus (T2D), the most common deleterious metabolic disease at present all over the world, is usually preceded by the combined effects of pancreatic β-cell dysfunction and insulin resistance^1^,^2^. Several lines of evidence suggest that insulin resistance is the key risk factor in the pathogenesis of T2D. Hence an accurate and early detection of insulin resistance is important to delay or prevent the acute onset of T2D. However, there is still a real challenge on when or how to evaluate individuals who are at high-risk for developing insulin resistance or during the preclinical phase of T2D.

To quantify the insulin resistance (i.e. inverse of insulin sensitivity), the hyperinsulinemic-euglycemic clamp (HEC) study has been considered over the decades to be the “gold standard” method. But due to several complications like continuous infusion of insulin, frequent blood glucose infusion and overall withdrawals of blood samples at different time intervals, it is difficult to apply in large-scale screening purposes^3^,^4^. Therefore, the different surrogate techniques such as the quantitative insulin sensitivity check index (QUICKI) and homeostasis model assessment (HOMA)^5^,^6^,^7^, derived primarily from the measurements of fasting blood glucose and fasting plasma insulin levels, are well accepted alternative methods for assessing insulin resistance^3^. More recently, another invasive surrogate index called insulin sensitivity index, ISI_0,120_, that exploits both the fasting (0 min) and post-dose (120 min) plasma insulin and blood glucose concentrations, has been proposed to be a viable method of whole-body insulin sensitivity for use in clinical settings^8^. The ISI_0,120_ is calculated by the following formula: 1/ISI_0,120_ = log mean insulin/MCR, where mean of 0 and 120 min blood glucose values are utilized to calculate the metabolic clearance rate (MCR)^9^. Results obtained by utilizing ISI_0,120_ were well correlated with the HEC study in comparison to the other surrogate sensitivity indexes. This is because of the fact that during oral glucose tolerance test (OGTT), the 0 and 120 min samples are more reproducible than other intermediate time points^10^. Moreover, HOMA-IR and QUICKI both estimate the hepatic glucose resistance, whereas the peripheral insulin resistance can be evaluated from the ISI_0,120_. Besides, the calculations for estimating HOMA-IR and other sensitivity indexes consider the insulin secretory capability, whereas ISI_0,120_ evaluates the insulin sensitivity in body. Nevertheless, the samples required for ISI_0,120_ measurement are less and it estimates better insulin sensitivity compared to the other sensitivity index formulas^8^. Therefore, the ability to non-invasively evaluate insulin sensitivity index (ISI_0,120_) for diagnosis of pre-diabetes and type 2 diabetes has a substantial clinical significance. Recently, it has been proposed that ^13^C-glucose breath test (^13^C-GBT) may be a non-invasive approach for quantifying insulin resistance by contrast with the direct invasive HEC study^5^. The ^13^C-GBT exploits the carbohydrate metabolism of orally administered ^13^C-labelled glucose substrate. The substrate is metabolized and produces ^13^C-labelled carbon dioxide (^13^CO_2_). This ^13^CO_2_ is then transported to the lungs through the blood stream, and finally it is excreted in exhaled breath. During OGTT, the glucose utilization for cellular fuel oxidation is strongly dependent on the insulin sensitivity. Postprandially, the alteration of insulin sensitivity in PD and T2D significantly decreases the cellular glucose uptake. In individuals with pre-diabetes and type 2 diabetes, when a dose containing isotopically labelled glucose [U-^13^C_6_] is ingested, the cellular response to this exogenous glucose is remarkably blunted. Individuals with insulin resistance or T2D will exhibit less carbon-13 isotopes of exhaled breath CO_2_ because of impaired glucose uptake by the cells^6^,^7^. However, the potential link between the exhaled breath ^13^CO_2_/^12^CO_2_ isotope ratios and ISI_0,120_ for the pre-diabetes and type 2 diabetes is not currently known. The ^13^CO_2_ / ^12^CO_2_ isotope ratios in the exhaled breath are usually expressed as delta-over-baseline (DOB) values described in the following way:


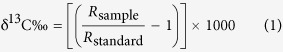



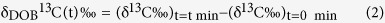


where R_sample_ and R_standard_ are defined as ^13^C/^12^C isotope ratio of the sample and international standard Pee Dee Belemnite (PDB) value (0.0112372) respectively. The isotope tracer, δ_DOB_^13^C(t)‰, quantified from the exhaled breath samples, can be followed to accurately evaluate the insulin resistance prior to the onset of T2D. Therefore, there is a pressing need to evaluate the clinical efficacy of the carbon isotopic fractionations of breath CO_2_ during the glucose metabolism for large-scale screening of individuals with insulin resistance and type 2 diabetes. Moreover, unravelling the potential link between the stable isotopes of carbon in breath CO_2_ and ISI_0,120_ may specifically track the pathogenesis of the preclinical phase of T2D and hence may introduce a new strategy for non-invasive evaluation of insulin resistance.

To find the association between the ^13^C/^12^C-isotope ratios of breath CO_2_ and the ISI_0,120_, we have analysed the exhaled breath carbon dioxide isotopes for the accurate and fast non-invasive assessment of insulin resistance in PD and T2D by means of a laser-based high-precision cavity-enhanced integrated cavity output spectroscopy (ICOS) system. In this article, we have demonstrated the associations between exhaled breath carbon-13 isotopes of CO_2_ and invasive parameters like blood glucose, insulin and HbA1c levels. Furthermore, we also determined several diagnostic parameters of the breath isotope analysis including sensitivity, specificity, optimal diagnostic cut-off points along with positive and negative predictive values to accurately evaluate the insulin resistance as well as the precise metabolic transition from normal to PD and then on to T2D.

## Results and Discussion

**Figure 1:**
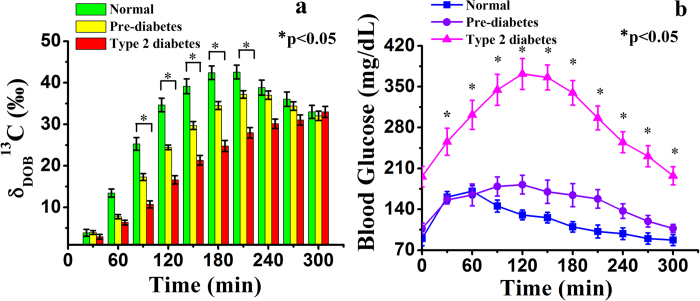
Kinetics study of breath carbon-13 isotope excretions and blood glucose levels after administration of ^13^C-glucose. **a**, Breath δ_DOB_^13^C (‰) enrichments. **b**, blood glucose concentrations for normal (NDC), pre-diabetes (PD) and Type 2 diabetes (T2D) individuals at every 30 min interval during 5 h-OGTT. *indicates statistically significant difference (p < 0.05). Data are expressed in terms of mean ± SEM.

We initially investigated the time-dependent excretion kinetics of stable carbon-13 isotopes, expressed as δ_DOB_^13^C(t)‰ values, in exhaled breath samples by ICOS method to investigate the distribution of ^13^CO_2_ isotopic abundance in breath samples associated with isotopically labelled glucose metabolism for NDC (non-diabetic control), PD and T2D and the results have been illustrated in Fig. 1a. We observed that the mean δ_DOB_^13^C(t)‰ values for the group with T2D were significantly lower (p < 0.01) compared to the groups with PD and NDC between 90 min and 210 min after the glucose load. These findings suggest that carbon isotopic fractionations of breath CO_2_ is capable of detecting marked differences in δ_DOB_^13^C(t)‰ values in exhaled breath samples within 90 min of a 5 h-OGTT among the groups with NDC, PD and T2D. However, there were no statistically significant differences (p > 0.05) in mean δ_DOB_^13^C(t)‰ values among all the groups from 240 min in response to glucose ingestion. We also simultaneously studied how the concentration of blood glucose changes with time in response to oral glucose ingestion.

Figure 1b indicates that blood glucose levels were significantly (p < 0.001) higher in T2D and PD groups compared with the NDC group, as expected. For an individual with T2D or PD, the impaired glucose uptake plays an important role for blunted glucose oxidation in cells because of diminished pancreatic insulin secretion or impaired insulin action on the target tissues^11^. The individuals with PD and T2D accordingly produce less amount of ^13^CO_2_ in exhaled breath samples compared with NDC. Our results support that the breath tests are more direct measure of intracellular glucose metabolism and impairments of exogenous (oral) glucose. Thus the monitoring of stable carbon isotopes may assist in non-invasive assessment of NDC, PD and T2D individuals by providing an alternative approach for large-scale screening purposes without the need for invasive repeated blood samplings.

**Figure 2:**
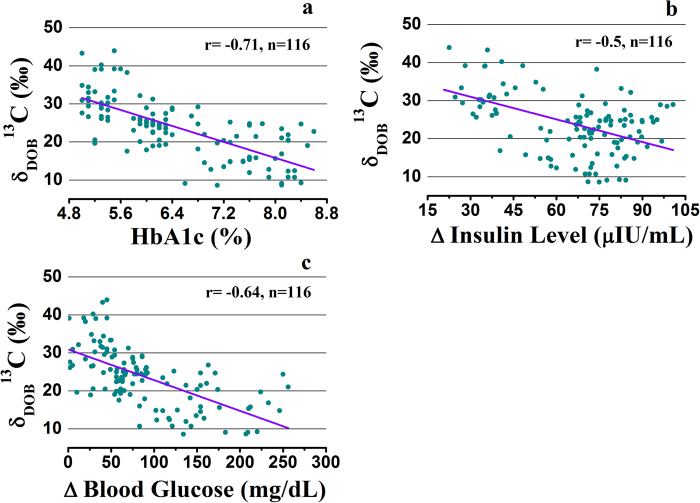
Linear regression plots to show the correlations of δ_DOB_^13^C (‰) in breath with different invasive parameters. **a**, breath δ_DOB_^13^C(‰) with glycosylated haemoglobin (HbA1c %). **b**, plasma insulin levels (∆ Insulin Level) from the baseline during 2 h-OGTT. **c**, shows correlation of blood glucose concentrations (∆ Blood Glucose). The data are statistically significant different (p < 0.001).

We next explored whether there were any correlations of δ_DOB_^13^C‰ values in exhaled breath at 120 min with the variables related to the insulin resistance, such as absolute changes in blood glucose levels, and plasma insulin levels at the particular time during the OGTT, in addition to glycated haemoglobin (HbA1c) measurements. Figure 2 depicts the inverse correlations of δ_DOB_^13^C‰ values with all these measured parameters. The correlation of δ_DOB_^13^C (t = 120 min)‰ with HbA1c (%) was strong with a correlation coefficient of r = −0.71 (p < 0.001) compared with the blood glucose (r = −0.64, p < 0.001) and plasma insulin levels (r = −0.5, p < 0.001).

**Figure 3:**
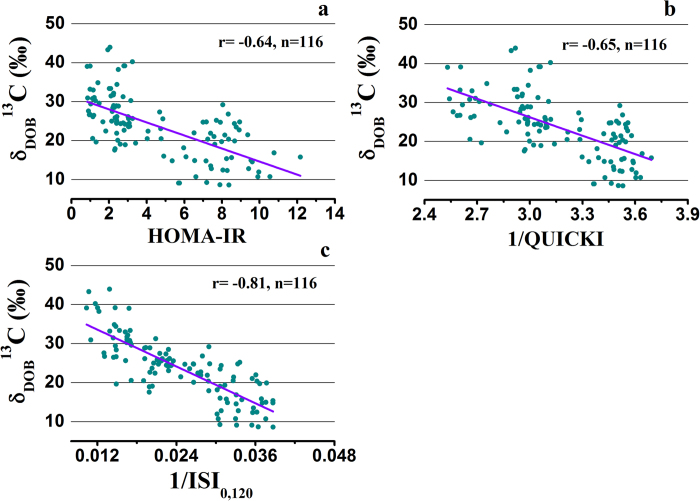
Correlations of δ_DOB_^13^C(‰) in breath with currently available insulin resistance assessment methods. **a**, HOMA-IR. **b**, 1/QUICKI. **c**, 1/ISI_0,120_. The data are statistically significant different (p < 0.01).

We further investigated the association of the δ_DOB_^13^C‰ values at 120 min in breath samples with the three surrogate methods of HOMA-IR, QUICKI and ISI_0,120_ to measure the insulin resistance in all individuals with different metabolic states. Significant inverse correlations (p < 0.01) between breath δ_DOB_^13^C‰ values and all the measured indexes were observed, as shown in Fig. 3. However, the best correlation was observed between δ_DOB_^13^C‰ values and 1/ISI_0,120_ (r = −0.81, p < 0.001), suggesting a significant association between the results of δ_DOB_^13^C‰ and ISI_0,120_ index.

Recent studies demonstrated that ISI_0,120_ is superior to HOMA-IR for the assessment of insulin resistance because of essentially two intrinsic drawbacks of HOMA model^12^,^13^. Firstly, HOMA index is based on the assumption that beta cell function is normal and therefore it can’t be applied for patients with type 2 diabetes where beta cell is destroyed in many cases. Secondly, HOMA-IR considers that the relationship between insulin and glucose is linear, but practically it is parabolic^1^. The QUICKI has also the similar disadvantages as like HOMA-IR^12^. Furthermore, during the OGTT, the majority of the insulin mediated glucose disposal takes place in peripheral tissues^14^, suggesting that the estimation of insulin resistance in peripheral tissue is more important to correlate with ^13^CO_2_ excretions from isotopically labelled glucose disposal for NDC, PD and T2D. It is noteworthy that HOMA-IR and QUICKI evaluate the hepatic insulin resistance essentially in fasting state, whereas ISI_0,120_ estimates the peripheral insulin resistance which is primarily responsible for exogenous glucose uptake. Therefore ISI_0,120_ may be the best predictor of insulin resistance and hence the correlation of δ_DOB_^13^C ‰ with ISI_0,120_ index is more practical to evaluate the efficacy of breath test to measure the insulin resistance.

**Figure 4:**
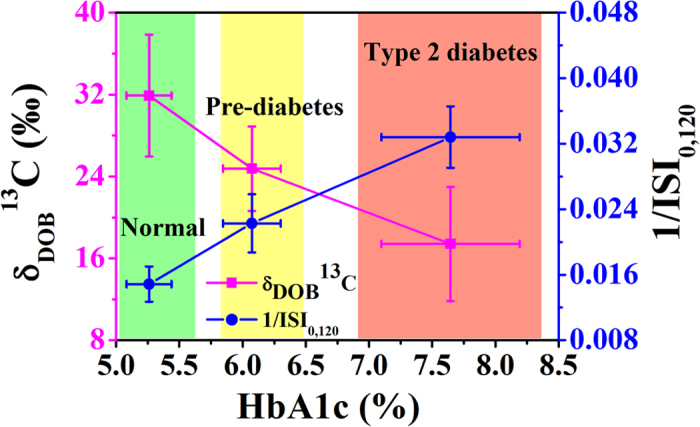
Distribution of δ_DOB_^13^C‰ and 1/ISI_0,120_ values against HbA1c (%) in normal (NDC), pre-diabetes (PD) and type 2 diabetes (T2D). Plot represents the clear transitions of δ_DOB_^13^C and 1/ISI_0,120_ from NDC (green) to PD (yellow) and T2D (red). Data are expressed as mean ± SD.

Taken together, these findings suggest that monitoring stable ^13^C/^12^C isotopes of breath CO_2_ in response to glucose ingestion may be an easy and non-invasive approach to evaluate the insulin resistance in individuals. In view of the present results, we posit that the measurements of ISI_0,120_ index as a surrogate marker of insulin resistance and thus may distinctively track the evolution of pre-diabetes prior to the onset of T2D, as depicted in Fig. 4. The distributions of δ_DOB_^13^C‰ and 1/ISI_0,120_ values illustrate that individuals can be classified into three distinct zones with NDC in green zone, PD (moderately insulin resistance) in yellow zone and T2D (sufficiently higher insulin resistance) in red zone.

**Figure 5:**
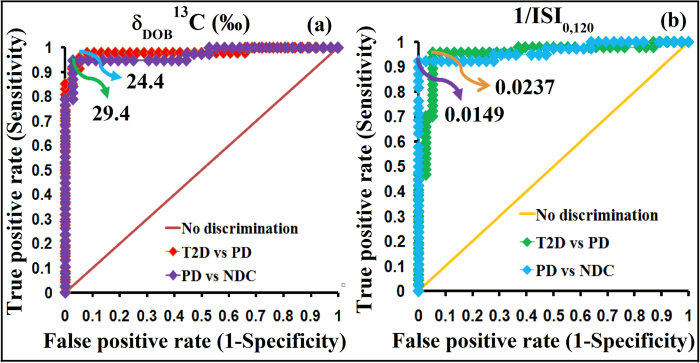
Receiver operating characteristic (ROC) curves to determine optimal diagnostic cut-off points. **a**, δ_DOB_^13^C‰ and **b**, 1/ISI_0,120_ for clinical diagnosis of normal (NDC), pre-diabetes (PD) and type 2 diabetes (T2D).

Finally, to investigate the precise metabolic transition from NDC to PD and then on to T2D, we determined the optimal diagnostic cut-off points of 1/ISI_0,120_ and δ_DOB_^13^C‰ values in exhaled breath using receiver operating characteristics curve (ROC) analysis (Fig. 5a and b). ROC curves were generated by plotting the true positive rate (sensitivity) against the false positive rate (1-specficity) using the values of 1/ISI_0,120_ and δ_DOB_^13^C‰. The highest values of sensitivity and specificity were used to calculate the optimal diagnostic cut-off points. A diagnostic cut-off point of 1/ISI_0,120_ = 0.0237 between individuals with PD and T2D, exhibited the sensitivity and specificity of 96.1% and 95.1%, respectively, whereas 1/ISI_0,120_ = 0.0149 accurately diagnosed individuals with NDC and PD. To apply breath analysis for the diagnosis of insulin resistant PD and T2D, we have calculated the optical diagnostic cut-off values of carbon-13 isotopes of exhaled breath CO_2_. We observed that individuals with δ_DOB_^13^C‰ < 24.4 and δ_DOB_^13^C‰ > 29.4 were considered as T2D and NDC respectively, whereas subjects with 29.4 > δ_DOB_^13^C‰ > 24.4 were suggested to be PD. These cut-off points corresponded to the similar levels of diagnostic sensitivity and specificity as shown in Table 1. We have calculated these cut-off values with 95% confidence intervals. Thus the analyses of stable isotopes of the major metabolite of human breath CO_2_ establish a broad clinical feasibility as a sufficiently robust non-invasive detection method for an accurate diagnosis of PD and T2D with different metabolic states of insulin resistance. We also finally explored the positive and negative predictive values (PPV and NPV) for the diagnostic assessment. These two parameters essentially indicate the probabilities of getting diseases once the actual test results of the patients are known^15^. The present method demonstrated diagnostic PPV of 98% between NDC vs PD, and 96% between PD vs T2D. It also exhibited diagnostic NPV of 94% between NDC vs PD, and 95% between PD vs T2D, indicating an excellent diagnostic accuracy for the accurate evaluation of insulin resistance in different metabolic states. However, it is important to note that the cut-off values may depend on the food habits in the different populations of various countries and the isotopic compositions of labelled glucose. We have determined the cut-off values based on the Indian populations utilizing the mentioned labelled glucose. Therefore, it would be interesting to estimate the cut-off values within the subject-variability and also to elucidate the probable dietary effects on breath isotope analysis in future studies.

## Conclusion

In conclusion, our study confirms the clinical feasibility of the exhaled breath carbon dioxide isotopes analysis for estimating insulin resistance and thereafter the diagnosis of non-diabetic control, pre-diabetes and type 2 diabetes. When ISI_0,120_ has been suggested as the most correlated alternative insulin resistance (1/insulin sensitivity) parameter with respect to euglycemic hyperinsulinemic clamp study, our observations demonstrated a strong correlation of ISI_0,120_ index with breath carbon dioxide isotopes, suggesting a new perspective into the non-invasive evaluation of insulin resistance rather than the traditional invasive measurements. Additionally, we first estimated the cut-off values of 1/ISI_0,120_ for the diagnosis of pre-diabetes and type 2 diabetes, thus making it a potentially robust approach for accurate evaluation of insulin resistance. Overall, our calculated optimal cut-off values of both 1/ISI_0,120_ and carbon-13 isotopes of breath CO_2_ also suggest that they may serve as useful methods for early detection and follow-up of individuals who are at high-risk for developing insulin resistance prior to the onset of type 2 diabetes that threaten modern society. Although, many important gaps may remain in understanding the potential link between ISI_0,120_ index and breath CO_2_ isotopes in the present study, our results, however, have significant implications in the isotope-specific molecular diagnosis of insulin resistant pre-diabetes and type 2 diabetes with broad clinical applications. Besides, this non-invasive approach for estimation of insulin resistance may assist in detecting the pre-diabetes stage of asymptomatic type 2 diabetic subjects in preclinical phase. This point-of-care diagnostic method may also help to overcome the current compliance of invasive techniques for screening diabetes mellitus in future days. Therefore, our proposed breath isotopes analysis may be a new method to prevent or treat the deleterious effect of the most common metabolic syndrome in the world. Finally, as this breath analysis approach is safe, simple and non-invasive, it could be an attractive option for large-scale screening purposes in a wide variety of individuals including children, pregnant women and seniors.

## Materials and Methods

### Subjects

A total of 116 human subjects (31 non-diabetic controls, 37 pre-diabetes and 48 type 2 diabetes) were recruited for the study. Individuals with hypertension, previous history of diabetes, taking any medication that may affect lipid or glucose metabolism, were excluded from the study. According to the guideline of the American Diabetes Association (ADA)^16^, the subjects were classified into three groups: non-diabetic controls with glycosylated hemoglobin (HbA1c %) <5.7 and 2-hr blood glucose < 140 mg/dL during the oral glucose tolerance test (2h-OGTT), pre-diabetes with 5.7 ≤ HbA1c (%) < 6.5 and 140 mg/dL ≤ 2-hr OGTT glucose < 200 mg/dL and type 2 diabetes with HbA1c (%) ≥6.5 and 2-hr OGTT ≥ 200 mg/dL. Individuals were selected from the treatment-naive population, where they were totally undiagnosed about the status of their diabetes. The clinical parameters are described in Table 2. The whole study protocol was approved by the Institutional Ethics Committee of Vivekananda Institute of Medical Sciences (Registration No. ECR/62/Inst/WB/2013) and the methods were carried out in accordance with the approved guidelines. Informed consent was collected from each participant according to the protocol approved by Institutional Ethics Committee of Vivekananda Institute of Medical Sciences. The study also received administrative approval by the S.N. Bose Centre, Kolkata (Ref. No: SNB/PER-2-6001/13-14/1769).

### Breath and blood samples collection and measurements

Subjects were asked for overnight fasting (~12 hours) before the study. A baseline breath sample was taken in a breath sample collection bag (QUINTRON, USA, SL No.QT00892), whereas blood sample was drawn from each participant in EDTA-vial. Thereafter, participants were instructed to ingest a test meal containing 75 mg U-^13^C_6_ labelled D-glucose (CIL-CLM-1396-CTM, Cambridge Isotope Laboratories, Inc., USA) along with the 75 g normal glucose dissolved in 150 mL water. From this starting point, the post-dose breath and blood samples were collected in every 30 minutes interval up to 5 h. Breath bags were designed in such a way that oral-breath first passed into a dead space and endogenously produced end-tidal breath entered into the reservoir bag through a one-way valve. During analysis, the breath samples were taken from the reservoir bags by Quintron air-tight syringe fitted onto the bag. Blood samples were utilized to measure the plasma glucose spectro-photometrically (2300 STAT Plus Glucose Analyzer) and insulin concentrations by using monoclonal antibody coated immunoassay DIA source INS-EASIA Kits (DIAsource ImmunoAssays, Belgium). Breath samples were analyzed by a high-resolution isotopic CO_2_ integrated cavity output spectrometer as described below. HbA1c (%) was measured by utilizing an HbA1c analyzer (D-10, USA) in HPLC method.

### Integrated cavity output spectrometer (ICOS) for breath analysis

We have employed a high-precision carbon dioxide isotope analyzer (CCIA 36-EP, Los Gatos research, USA) exploiting integrated cavity output spectroscopy (ICOS) to analyze the isotopic compositions of CO_2_ in exhaled breath samples. The technical details and its working principle have been described elsewhere^17^,^18^,^19^. In brief, the ICOS system consists of two high reflectivity mirrors (R ~ 99.98%) placed at the two ends of a high-finesse optical cavity (59 cm long). A continuous wave (cw) diode laser (distributed feedback) operating at near infrared ~2.05 μm was utilized to scan over 20 GHz to record the absorption features of ^12^C^16^O^16^O and ^13^C^16^O^16^O isotopes at the wave numbers of 4874.448 cm^−1^ and 4874.086 cm^−1^ respectively, in the (2,0^**0**^,1)←(0,0^**0**^,0) vibrational combination band of the CO_2_ molecule. The laser light moves back and forth inside the optical cavity and provides an effective optical path length of ~3 km. The measurement cell of ICOS instrument was maintained at a temperature of 46 °C by a resistive heater and feedback control system. The typical pressure of the measurement cell was ~30 Torr which was achieved by a diaphragm pump. The real-time ro-vibrational spectral features were fitted with Voigt profiles and the absolute concentrations of ^12^C^16^O^16^O and ^13^C^16^O^16^O isotopes in exhaled breath samples were determined using Beer’s law. The ICOS system was calibrated by the three standard certified gases of 5% CO_2_ in air with different δ^13^C‰ values (δ^13^C‰ = −22.8‰, −13.22‰ and −7.3‰, Cambridge Isotope Laboratory, CIL, USA). The accuracy and precision of the δ_DOB_^13^C‰ using the ICOS system were in the range of 98–99% and 0.15‰, respectively.

### Insulin resistance index

HOMA-IR, QUICKI and ISI_0,120_ were calculated from the blood glucose and insulin levels in the fasting (0 min) and post-dose (120 min) conditions. Insulin resistance indexes obtained from insulin and glucose concentrations during 2 h-OGTT study and these were measured by utilizing the following equations^9^:


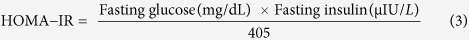



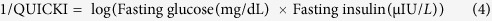



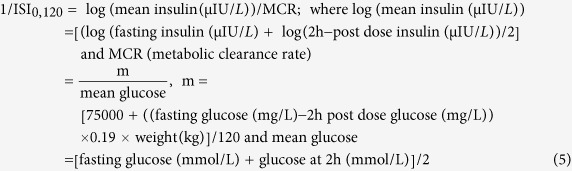


### Statistical Method

Data were usually expressed as mean ± SE. To compare the non-parametric distribution, we applied Mann-Whitney and Kruskal-Wallis tests, whereas the normal distributed data were analyzed by one way analysis of variance (ANOVA). To determine the optimal cut-off points, we utilized receiver operating characteristic curve (ROC)^20^ analysis. The optimal cut-off value was considered as the cut-off point where we obtained maximum diagnostic sensitivity (true positive rate) and specificity (true negative rate). Data were analyzed by Origin Pro 8.0 (Origin Lab Corporation, USA) and Analyse-it Method Evaluation software (Analyse-it Software Ltd, UK, version 2.30). A two-sided p values < 0.05 was considered as statistically significant. Here, p values have been calculated to check the differences among the three individual groups.

## Additional Information

**How to cite this article**: Ghosh, C. *et al.* Insulin sensitivity index (ISI_0, 120_) potentially linked to carbon isotopes of breath CO_2_ for pre-diabetes and type 2 diabetes. *Sci. Rep.* **5**, 11959; doi: 10.1038/srep11959 (2015).

## Figures and Tables

**Table 1:** Determination of important diagnostic parameters related to cut-off values of 1/ISI _0,120_ and δ_DOB_^13^ C (‰) by ICOS method for screening individuals with non-diabetic control (NDC), pre-diabetes (PD) and type 2 diabetes (T2D).

Groups	Cut-off points of δ_DOB_^13^C‰	Sensitivity	Specificity	PPV	NPV	AUC	Accuracy
NDC vs PD	29.4	94.7%	97.2%	97.3%	94.6%	0.97	95.9%
PD vs T2D	24.4	97.9%	92.1%	93.8%	97.2%	0.98	95.3%
							
**Groups**	**Cut-off points of** 1/Ι**SI**_**0,120**_	**Sensitivity**	**Specificity**	**PPV**	**NPV**	**AUC**	**Accuracy**
NDC vs PD	0.0149	92.1%	100%	100%	92.3%	0.963	95.9%
PD vs T2D	0.0237	95.7%	95.7%	95.7%	94.7%	0.954	95.3%

AUC: area under the curve; PPV: positive predictive value; NPV: negative predictive value.

**Table 2:** Clinical characteristic of the study subjects.

Parameters	Non-diabetic control (NDC) (n = 31)	Pre-diabetes (PD) (n = 38)	Type 2 Diabetes (T2D) (n = 47)	p values
Sex (M/F)	18/13	26/12	31/16	
Age (Years)	35.48 ± 6.8	34.55 ± 6.6	38.63 ± 9.8	0.632
Weight (kg)	65.01 ± 6.5	65.93 ± 7.1	65.92 ± 5.6	0.789
BMI (kg/m ^**2**^)	24.43 ± 2.7	24.21 ± 2.5	23.93 ± 2	0.807
Fasting Plasma Glucose (mg/dL)	90.1 ± 12.5	107.2 ± 9.1	196 ± 17.6	<0.001*
Fasting Plasma Insulin (μIU/mL)	7.57 ± 3.5	10.51 ± 2.6	22.94 ± 4.1	<0.001*
2-hr Post-dose Plasma Glucose (mg/dL)	144.3 ± 11.2	182.1 ± 16.1	372.1 ± 26.5	<0.001*
HbA1c (%)	5.26 ± 0.2	6.07 ± 0.2	7.64 ± 0.5	<0.001*
HOMA-IR	1.68 ± 0.76	2.79 ± 0.8	7.92 ± 1.5	<0.001*
1/QUICKI	3.49 ± 0.08	3.04 ± 0.11	2.79 ± 0.19	<0.001*
1/ISI _**0,120**_	0.014 ± 0.002	0.023 ± 0.002	0.046 ± 0.012	<0.001*

*Represents statistically significant differences among non-diabetic control (NDC), pre-diabetes (PD) and type 2 diabetes (T2D). M and F stand for male and female, respectively. *p < 0.05 (statistically significant). Results are mean ± SD.
